# The first Portuguese Open Dialogue pilot project intervention

**DOI:** 10.3389/fpsyg.2023.1175700

**Published:** 2023-09-07

**Authors:** Sofia Tavares, Joana Ribeiro, Sofia Graça, Bruna Araújo, Mariana Puchivailo, João G. Pereira

**Affiliations:** ^1^Department of Psychology, Évora University, Évora, Portugal; ^2^Department of Psychology, CIEP, University of Évora, Évora, Portugal; ^3^Instituto Universitário de Ciências Psicológicas, Sociais e da Vida (ISPA)—Instituto Universitário, Lisbon, Portugal; ^4^Romão de Sousa Foundation, Estremoz, Portugal; ^5^Department of Psychology, FAE University Center, Curitiba, Paraná, Brazil

**Keywords:** Open Dialogue, psychiatric crisis, dialogic practice, democratic approaches, mental health care

## Abstract

**Introduction:**

In 2020, the Directorate General of Health (DGS), a central service of the Ministry of Health in Portugal, approved and co-financed the first Open Dialogue program in the country. The present report aims to demonstrate the preliminary results of the first year of the project, implemented in the northern interior region of Alentejo.

**Methods:**

Seven people at the Center of Concern (PCC) and 21 family members/social networks received care through Open Dialogue; four external social workers and psychologists were also involved in the project as members of the support network. A total of 160 network meetings were undertaken, reaching as many as 27 per month in the busiest periods. Based on a previous Italian Research Protocol, developed by Pocobello et al. (non-published manuscript), quantitative and qualitative data were collected in and after the clinical meetings involving PCC and their family/social network, through a multi-method approach: clinical history interview (e.g., generic research on sociodemographic data, duration of untreated symptoms, reasons for requesting help, possible hospitalizations, and/or treatments/therapies) and the following scales applied every five sessions (e.g., CORE-OM, BSI, GAF, and LSNS-6).

**Results:**

The preliminary results indicate an improvement in global functioning and the enlargement of social network size/support, a decrease in symptoms, and a negative correlation between the number of sessions and the LSNS6. Medication use remained largely unchanged at the end of the project.

**Discussion:**

In general, even with a small sample, the results are considered satisfactory and seem to be aligned with the vast majority of Open Dialogue studies, which for several decades have consistently pointed toward better recovery rates than treatment as usual as well as increased client satisfaction. We expect that the results presented can boost further research and help strengthen the OD approach.

## 1. Introduction

Open Dialogue (OD) is a Finnish therapeutic approach and an organizational system of mental health services aimed at responding to psychiatric crises. OD was inspired by the need-adapted treatment of Alanen (1997, 2009) and based on psychodynamic therapy, family therapy, dialogical practices, and network approaches. Efforts were undertaken to allow for an immediate response at the onset of a psychotic crisis. This study aimed to create a psychotherapeutic and dialogical space—particularly within the so-called network meetings—where the Person at the Center of Concern (PCC) would participate together with his/her family and/or support network. Priority is given to transparent and shared decision-making in a dialogical format (Altonen et al., 2011).

Open Dialogue has faced several adaptations according to different contexts and countries. Nevertheless, a set of principles remain central to the accurate implementation of OD practice: immediate help, social network perspective, responsibility, flexibility, mobility, tolerance of uncertainty, and dialogism (Seikkula and Olson, 2003; Pereira et al., 2019). The team aimed to create a therapeutic space that tolerates uncertainty while letting understanding unfold from multiple perspectives, allowing for natural resolution when possible (Olson et al., 2014). Treatment plans and decisions are made in co-participation and transparently.

OD has been systematically evaluated over the last three decades (Lakeman, 2014; Freeman et al., 2019; Kantorski and Cardano, 2019; Cooper et al., 2020), showing promising results regarding returning to work and/or academic activities (Seikkula et al., 2006, 2011; Altonen et al., 2011; Alakare and Seikkula, 2022), reduction of psychiatric symptoms (Gordon et al., 2016), relapses (Seikkula et al., 2011), days of hospitalization (Altonen et al., 2011; Bergström et al., 2018), use of anti-psychotic medication, and allocation of disability and unemployment benefits (Bergström et al., 2018; Alakare and Seikkula, 2022). Poor quality social networks and delays in assistance during psychiatric crises lead to a higher frequency of hospitalizations and a propensity for anti-psychotic medication use (Seikkula et al., 2001).

OD presents itself as an alternative to the traditional perspectives based on the problem–diagnosis–treatment triad (von Peter et al., 2019). It is currently recognized by the World Health Organization (WHO) as good practice in psychiatric crisis, as well as a recovery and human rights supporter. It is also present in the Council of Europe's good practice compendium, whose purpose is to eradicate coercive practices in mental health settings (Mosse et al., 2023). This reinforces OD's alignment with human rights—a worldwide concern in the context of mental health (von Peter et al., 2019).

Portugal was already known as one of the European countries with the highest prevalence of mental disorders (Direção Geral da Saúde—DGS, 2017) and, according to the data collected in 2020, the country was classified with the highest prevalence (23%) of symptoms associated with psychological issues (Entidade Reguladora da Saúde—ERS, 2023) as well as one of the highest uses of psychotropic drugs in the EU (Almeida et al., 2013). There are serious difficulties in the identification, treatment, and follow-up of adults with mental disorders, which is reflected in the excessive use of hospital emergencies and the high rate of involuntary hospitalizations (ERS, 2023).

These problems are also a consequence of the scarcity and lack of human resources in psychology and psychiatry. The current number of psychologists is far below the recommended ratio of 1 psychologist per 5,000 inhabitants, currently at 1 per 9,687 inhabitants (Ordem dos Psicólogos Portugueses—OPP, 2022). This problem anticipates constraints in accessing psychological help.

Alentejo exhibits the highest ratio of depression, anxiety, and suicide (ERS, 2023) in the country. A total of 5.4% of the population is illiterate, compared with 3.8% in the rest of the country. It is the region with the lowest population density, the highest aging/longevity index, and one of the highest unemployment rates in the country (Instituto Nacional de Estatística—INE, 2022b).

In this context, OD should be seen as a new (respectful) way of understanding and responding to mental health problems, accessible to the Portuguese health system.

The research protocol for assessing the transferability of the OD approach to the context of North Alentejo mental health services included different levels of evaluation: (1) perceptions of the mental health service managers of the region; (2) evaluation of the impact of OD training in the Romão de Sousa Foundation clinical team, as well as in its clinical practice; (3) adherence evaluation; and (4) therapeutic outcomes.

The Romão de Sousa Foundation set up a small OD crisis team composed of two clinical psychologists with an advanced specialty in psychotherapy, one clinical psychologist with a PhD in psychotherapy—coordinator, and one psychiatrist. They were all trained in Open Dialogue up to the practitioner level, with training in Finland, Norway, the United States, and Portugal. The external supervision during the project was undertaken by Professor Mary Olson from Yale University and the Institute for Dialogic Practice in the United States. The team operated 5 days a week with the aim of improving the quality of services (psychiatric, psychological, and social) for the population in severe mental distress and the psychosocial and socio-professional empowerment and capacity building of people in the center of concern. Throughout the program, all the procedures that ensure the fidelity of OD practices were adopted, such as video recording of all sessions for supervision and audit purposes.

The present study aimed to analyze the preliminary clinical results of the first year of the Portuguese Open Dialogue program implemented in the northern interior of the Alentejo region. We would like to know whether the results of the program follow the international trend of OD results, particularly regarding the improvement in participants' global functioning and the reduction of psychopathological symptoms. We also want to know whether certain sociodemographic variables (e.g., social network support) are related to clinical outcomes.

## 2. Materials and methods

### 2.1. Study design

This exploratory study is a naturalistic observational cohort of consecutive referrals of clients with psychiatric diagnoses treated with the OD approach. A prospective follow-up design was used, comparing baseline scores of client-level outcomes at every five sessions for 12 months.

### 2.2. Sample

In the initial sample, there were 11 eligible participants. However, due to the loss of interest and/or incompatibilities with the modality of the meetings, which were forced to be online due to COVID-19 confinement, the final sample ended with seven participants.

### 2.3. Sociodemographic and clinical characterization

Study participants had to be aged between 14 and 65 years, experiencing psychotic symptoms or other diagnoses of severe mental disorders, presenting for emergency services voluntarily, able to provide informed consent, and willing to have family and other social networks participate in the meetings. The final sample was composed of seven participants, five were female participants (71.4%) and two were male participants (28.6%); four of them were employed and/or in training, two were unemployed (one short-term and one long-term), and one was retired.

Regarding hospitalization, only one participant (14.3%) referred to being in a hospital or other residential structure before joining the Open Dialogue Project. Concerning suicide attempts, three participants (42.9%) declared having attempted suicide, and one participant (14.3%) presented self-harm behavior. Regarding medication, six (85.7%) participants were under psychiatric prescriptions at the onset, undertaken by professionals external to the treatment/research team, more precisely professionals who accompanied the participants before entering the project. After enrolling for OD treatment, it was the OD team psychiatrist that took responsibility for any changes in medication, in line with the characteristic OD joint decision-making during network meetings. There was only one exception in which the previous psychiatrist retained prescription responsibility and was invited to network meetings. Concerning extra-familial social relationships, six participants reported that they did not feel satisfied with their social relationships.

### 2.4. Procedures of participant screening and enrollment

Data were collected through non-random (objective) sampling. The OD treatment clients (and then the study participants) were recruited among clients who have access to mental health services at different levels (e.g., inpatient ward and mental health crisis service), through community structures such as the Commission for the Protection of Children and Young People (CPCJ), the Centre for Family Support and Parental Counselling (CAFAP), the Norte Alentejano Local Health Unit (ULSNA); leaflets distributed in Pharmacies, Social Centers, Town Hall; Internet; and Casa de Alba Therapeutic Community. Referrals were largely undertaken by the applicant's family and extended network or by the applicant himself or herself. All the participants who voluntarily agreed to participate in the OD treatment also consented to be part of the research sample. However, OD treatment and research were independent and required separate consent forms, so it was not mandatory to participate in both to be eligible for OD treatment. The participants were diagnosed with several disorders, such as anxiety disorders, affective disorders, psychotic disorders, and other situations such as suicidal ideation, emotional dysregulation, severe difficulties in relationships and in maintaining daily activities, and a moderate or high degree of psychosocial disability resulting from mental health problems. The diagnoses were not carried out by the OD clinical team but by clinicians from public or private health services who previously had contact with the participants.

The eligibility criteria for OD pilot project participants were being aged between 14 and 65, experiencing psychotic symptoms or other severe mental disorder diagnoses, voluntarily presenting to emergency services, being able to provide informed consent, and willing to have family and other social networks participate in the meetings. Members of the clinical staff were instructed about screening potential participants and evaluated to determine whether they were eligible for the study. Clinicians informed eligible clients about the possibility of taking part in the study and, when possible, registered reasons for eventual refusals. Once the participants had signed the informed consent, the enrollment was considered complete. The OD clinical and research teams were independent, except for the coordinator and last author of this article, who has been involved in both; however, most of the research team members have held or are holding positions at the Romão de Sousa Foundation, the institution that ran the clinical project. After accepting to participate in the research, the participants filled out the proposed questionnaires to monitor the process. The questionnaires, applied by the OD clinical team, were planned to be repeated every five sessions, but collection procedures became more complex with the start of the COVID-19 pandemic, and we only used data from baseline and after treatment. As informed by OD principles, no meeting frequency and/or treatment plans were imposed in advance. Instead, it was jointly decided throughout each meeting according to each participant's needs. Due to the COVID-19 pandemic, the program setting was forcedly adapted to the needs of the context with most contacts, and so OD meetings and assessments were performed remotely from the second month of the project onward, despite being initially designed to take place in a location of the participant's preference. At the end of treatment, 151 meetings were held online (94%) and only nine meetings (6%) were held in person.

This research was reviewed and approved by the Ethics Committee of the *Universidade de Évora*.

### 2.5. Instruments

OD feasibility was assessed through a set of quantitative and qualitative data, collected in/and after the clinical meetings involving people in the center of concern and their families/caregivers, through a multi-method approach: clinical history interview (e.g., generic research on sociodemographic data, duration of untreated symptoms, reasons for requesting help, possible hospitalizations, and/or treatments/therapies) and the following self-report scales applied every five sessions: CORE-OM (Sales et al., 2012, original from Evans et al., 2002), BSI (Canavarro, 1999, original from Derogatis and Spencer, 1982), GAF (Endicott et al., 1976), LSNS-6 (Ribeiro et al., 2012, original from Lubben et al., 2006), and a Satisfaction questionnaire. In this article, we only present part of these data, namely the ones related to the participants' sociodemographic and clinical characterization, and their therapeutic outcomes.

Regarding the instruments, the Clinical Outcomes in Routine Evaluation—Outcome Measure (CORE-OM) instrument consists of 34 items, on a scale from 0 to 4, distributed by wellbeing, problems/symptoms, life functioning, and risk to self and others domains, measuring psychological distress and essential aspects of psychological wellbeing over the last week (Sales et al., 2012). The cutoff is 1.25, with higher scores meaning greater severity of symptoms and distress.

The Portuguese Brief Symptom Inventory (BSI) is a self-assessment questionnaire, referring to the last week and consisting of 53 items, on a scale from 0 to 4, including nine dimensions: somatization, obsession-compulsion, interpersonal sensitivity, depression, anxiety, hostility, phobic anxiety, paranoid ideation, and psychoticism. The scale seeks to provide summary indices of the levels of psychopathological symptoms (Canavarro, 1999). The higher the scores, the greater the degree of symptomatology.

The Global Assessment of Functioning (GAF) Scale is divided into 10 sections and aims at assessing the impairment caused by mental disorder in psychological symptoms, social, and occupational functioning, i.e., how much individual symptoms affect daily life, on a scale from 0 to 100, with a 100 score evidencing superior functioning with no symptoms that impair functioning; from 61 to 70 some mild symptoms (e.g., depressed mood and mild insomnia), or some difficulty in social, occupational, or school functioning but generally functioning well, with some meaningful interpersonal relationships; from 41 to 50 serious symptoms (e.g., suicidal ideation, severe obsessional rituals, and frequent shoplifting) or any serious impairment in social, occupational, or school functioning; and scores below 21 as some danger of hurting self or others (e.g., suicide attempts without clear expectation of death, frequently violent, and manic excitement) or occasionally fails to maintain minimal personal hygiene or gross impairment in communication (Endicott et al., 1976).

The Lubben Social Network Scale (LSNS-6; Ribeiro et al., 2012) aims to assess people's social isolation and obtain information about the type of social relationships, the size of the network, and the intimacy with support network members. The LSNS-6 consists of six items distributed in two subscales, the Family subscale and the Friends subscale. The scale scores range from 0 to 30 on a 5-point Likert scale.

The satisfaction questionnaire was measured on a 0 to 10 scale.

### 2.6. Data analysis

A paired samples *t*-test was run for the preliminary exploration of the GAF, BSI, CORE-OM, and LSNS6 general clinical outcomes, which included data from baseline and the end of therapy. The Shapiro–Wilk test was run to verify normality distribution. Furthermore, we ran a series of bivariate correlations (Pearson's) among the variables age, number of meetings, satisfaction, program duration, and the scores of the last period of the clinical instruments. For the statistical data analysis, IBM-SPSS 28.0 was used.

## 3. Results

Table 1 summarizes the survey results, showing the scores for the GAF, BSI, CORE-OM, and LSNS6, including the baseline and final period results, as well as statistical data.

**Table 1:** Paired samples *t*-test for GAF, BSI, CORE-OM. and LSNS6.

	***M* (baseline)**	**SD (baseline)**	***M* (final)**	**SD (final)**	***t***	**df**	***p***	**Hedges' G**
GAF	57.71	10.468	65,71	11.398	−2.506	6	0.023	0.887
BSI	1.585	0.744	1.078	0.350	1.921	6	0.052	0.631
CORE-OM	1.899	0.883	1.252	0.343	1.712	6	0.069	0.562
LSNS6	12.429	5.533	13.429	4.315	−0.548	6	0.302	−0.194

GAF, Global Assessment of Functioning; BSI, Brief Symptom Inventory; CORE-OM, Clinical Outcome Routine Evaluation—Outcome Measure; LSNS, Lubben Social Network Scale; M, mean; SD, standard deviation; *t*, student's *t*-distribution; *df*, degrees of freedom; α = 0.05; *p* (one-tailed).

The GAF test results showed that the participants' scores of global functioning increased from baseline (*M* = 57.71; SD = 10.468) to the last period (*M* = 65.71; SD = 11.398); [*t*_(6)_ = −2.506; *p* = 0.023; g = −0.887), with statistical significance evidence and Hedges' g large effect.

The BSI test results showed that the participants' pathological symptomatology scores decreased from baseline (*M* = 1.585; SD = 0.744) to the last period (M = 1.078; SD = 0.350); [*t*_(6)_ = 1.921; *p* = 0.052; *g* = 0.631), marginally non-significant statistically.

The CORE-OM test results showed that the participants' psychological distress symptom scores decreased from baseline (*M* = 1.899; SD = 0.883) to the last period (*M* = 1.252; SD = 0.343); [*t*_(6)_ = 1.712; *p* = 0.069; *g* = 0.562], which was non-significant statistically.

The LSNS-6 test results showed that the participants' social network size/support increased from baseline (*M* = 12.429; SD = 5.533) to the last period (*M* = 13.429; SD = 4.315); [*t*_(6)_ = −0.548; *p* = 0.302; *g* = −0.194], even so, non-significant statistically.

Table 2 summarizes the outputs of the bivariate correlations among the variables age, number of meetings, satisfaction, program duration, and the scores of the last period of the clinical instruments.

**Table 2:** Pearson's correlations among the variables under study.

	**1**	**2**	**3**	**4**	**5**	**6**	**7**	**8**
Age	–							
Number of meetings	0.440	–						
Satisfaction with programme^a^	0.223	0.014	–					
Programme duration^b^	0.355	0.579	0.590	–				
GAF^c^	−0.323	−0.083	0.041	0.537	–			
BSI^c^	0.160	0.402	−0.661	−0.104		–		
CORE-OM^c^	0.647	0.177	−0.096	−0.131			–	
LSNS6^c^	−0.574	−0.896^*^	0.018	−0.402				–

GAF, Global Assessment of Functioning; BSI, Brief Symptom Inventory; CORE-OM, Clinical Outcome Routine Evaluation—Outcome Measure; LSNS, Lubben Social Network Scale.

a Mean scores.

b Months.

c Final scores.

* *p* < 0.01.

A very strong negative correlation between the number of sessions and the LSNS6 score was found (*r* = −0,896; *p* = < 0.01).

The participants' satisfaction mean score was 9.5 on a scale from 0 to 10, with 10 being the best score. Additionally, some of them expressed words of gratitude regarding the support received by the OD clinical team, e.g.,: *I'm feeling a lot of support; “I'm very reserved and quiet and you manage to get me to talk a little and bring out some problems that affect me the most; I really like the support of the whole team. It has been a great help for me and the family to overcome the difficulties we are experiencing; I really like the team, they helped me a lot; I hope they keep up the good work they do and help more people who need help like I did; Commitment in helping others solve problems”*.

At the end of the program, psychiatric prescriptions were kept by the participants who were using them at the beginning.

## 4. Discussion

According to the data, the OD approach presents favorable results, showing increased levels of global functioning and social network, as well as decreased symptomatology. The increment of GAF scores from moderate symptomology (from 51 to 60; usually with a predominance of flat affect and difficulties in social, occupational, or school) to a higher range score (from 61 to 70), evidence of less severe symptomatology (as depressed mood and mild insomnia), a tendency to improve personal relationships, and a positive level of functioning. Furthermore, one of the participants who was on medical leave returned to work, and another enrolled in university and joined OD training as a peer. These individual examples seem to sustain the quantitative measures that indicate functional improvement. Along with these results, we also observed an enlargement of the social network size, although it was residual. The family's and/or social network's participation in the dialogic process is highly encouraged due to their potential to become active allies, and their participation is expected to increase mutual understanding. The tendency to earlier relapses in people with low socialization levels is known (Johnstone et al., 1992, cite in Seikkula et al., 2001), even when among the first episodes of psychiatric crises the network size was found to be similar to that of the non-clinical population.

Efforts must continue to try to guarantee that factors such as social and family meaningful interactions are not neglected due to their importance to the recovery process and relapse prevention (McFarlane, 2016; Day and Petrakis, 2017; Johansen et al., 2021).

BSI and CORE-OM scores also decreased at the end of the project, which indicates that participants were under less psychological distress and more able to experience wellbeing, although other factors may have contributed to this outside therapy. A very strong negative correlation was found between the number of sessions attended and the LSNS6 final score, and due to our small sample size, we easily realized that the participants who attended more OD meetings scored lower on the LSNS6 at the end of the program. Although correlation does not imply causation, this result makes us wonder, once more, about the importance of family/social support and how the OD team might have, in some way, replaced the ones who were not available (or did not even exist). Although challenging, it is relevant to project how services can be adapted to the singular reality of each person looking to decrease perceptions of lack of support and improve integration into the community in a sustainable and fulfilling way. The data will be further analyzed to search for other possibly meaningful interactions. Follow-up outcomes are expected, so more conclusions about the OD's long-term outcomes can be reported.

As limitations of this research, we highlight the small sample size and the constraints due to the COVID-19 pandemic, which impact the adherence and retention of the participants, as the meetings were forcedly migrated online (94%), and some of them were not able to meet certain technological needs.

In Portugal, 26.6% of the population aged 16 or over reported a negative effect of the COVID-19 pandemic on mental health in 2021 (INE, 2022a). As the project occurred during the first year of the pandemic, we wonder about the possible influences it may have had on our sample and the consequent impact on outcomes, despite not being assessed. The non–self-report measure (GAF) was applied by all members of the clinical team present in the network meeting, rated blindly and immediately after the session, with the lowest number being recorded. The aim of using GAF was to increase the confidence of the self-reported measures by analyzing whether there was concordance between them. Satisfaction was not assessed; neither for network support members nor the clinical team. In addition, family/social relationship satisfaction was not assessed post-intervention. Furthermore, a more comprehensive satisfaction questionnaire would have provided better insight into the aspects valued by participants during the process.

Another limitation was the impossibility of getting access to Treatment as Usual results from the local health authority so that a comparison could be made. ERS (2023) identifies the need for improving IT systems, which currently lack systematization of information regarding the registration and control of health system beneficiaries. This aspect seems essential to an effective characterization of the population and follow-up procedures.

Despite the alarming facts regarding the higher incidence of mental health problems in this region, it is worth mentioning that efforts are being made to counter the rooted lack of investment in mental health in Alentejo.

In general, even with a small sample and with the limitations presented, the results seem to be aligned with the vast majority of Open Dialogue studies, which for several decades have consistently pointed toward better recovery rates than Treatment as Usual results as well as increased client satisfaction. Although not all the mean differences were statistically significant, these preliminary results are considered satisfactory, and agreeing with the fact that there is still much to be explored about OD and the transformations that its practice can bring (Mosse et al., 2023), we expect that the results presented here can boost further research and help strengthen the OD approach.

We also speculate that future Open Dialogue studies could include participants with generic mental health problems and not just psychosis as most Open Dialogue studies have performed so far.

Finally, we believe that future clinical trial results will help clarify the benefits of Open Dialogue and help give meaning and significance to small reports of this kind.

## Data availability statement

The original contributions presented in the study are included in the article/supplementary material, further inquiries can be directed to the corresponding author.

## Ethics statement

The studies involving humans were approved by Ethics Committee of Évora University. The studies were conducted in accordance with the local legislation and institutional requirements. Written informed consent for participation in this study was provided by the participants' legal guardians/next of kin. Written informed consent was obtained from the individual(s), and minor(s)' legal guardian/next of kin, for the publication of any potentially identifiable images or data included in this article.

## Author contributions

JP and ST provided guidance and supervision for the process. All authors contributed to the article and approved the submitted version.
